# Resequencing the Genome of *Bifidobacterium breve* Strain CECT7263

**DOI:** 10.1128/genomeA.00299-17

**Published:** 2017-05-04

**Authors:** Noelia Martínez, Roberto Luque, Mónica M. Olivares, Abelardo Margolles, Oscar Bañuelos

**Affiliations:** a Dairy Research Institute of Asturias, Spanish National Research Council (IPLA-CSIC), Villaviciosa, Asturias, Spain; b Research Department, Biosearch S.A., Granada, Spain

## Abstract

The probiotic properties of *Bifidobacterium breve* CECT7263, as well as its safety, have been the focus of in several studies since 2008, including the sequencing of its genome in 2012. This study aims to complete the available genomic data to deepen the knowledge of some phenotypic characteristics of this strain.

## GENOME ANNOUNCEMENT

*Bifidobacterium breve* is one of the bacterial species usually recognized as probiotics. Its presence in the gut flora, and therefore its consumption, is associated with benefits in several aspects of the health of the host (1), which is why there is an increasing number of food products containing bifidobacteria, mainly in the field of dairy and infant nutrition (2). The assertion of safety of probiotic strains requires the determination of their susceptibility to antibiotics and the characterization of possible mechanisms of resistance (3).

Preliminary studies showed that *B. breve* CECT7263 was resistant to three clinically relevant antibiotics: streptomycin, clindamycin, and erythromycin (4). Analysis of the genome sequences published in 2012, grouped in 34 contigs ([5]; GenBank accession no. AFVV00000000) revealed a point mutation in the *rsp*L gene, which is involved in streptomycin resistance in other *Bifidobacterium* strains (6). However, no gene related to erythromycin and/or clindamycin resistance was identified after revision of the available DNA sequences. At this point, we decided to perform a new *de novo* sequencing and genome annotation study.

The new draft genome sequence of *B. breve* CECT7263 was determined using a 251-bp paired-end library with Illumina MiSeq technology (Illumina, USA) at GenProbio SRL (Parma, Italy). A total of 423,772 reads were generated and assembled into 25 contigs using MIRA version 4.0.2 and the following tools in order to refine the final sequences: the Burrows–Wheeler aligner, SAMtools suite, VarScan version 2.2.3, and GATK software package version 2.8-1. Offline open reading frame (ORF) prediction was performed with Prodigal version 2.6 and its automatic annotation by means of a BLAST comparison against the NCBI databases and HMMER against the PFAM database. To identify sequences corresponding to rRNA and tRNA genes, RNAmmer version 1.2 and tRNAscan-SE version 1.21 were employed, respectively. The new draft genome sequence is composed of 2,330,408 bp, which contains 1,951 ORFs (2,314,396 and 1,868 ORFs in the previous genome sequence) and a G+C content of 58.9%. *In silico* analyses with genome sequence revealed the absence of plasmid replicon sequences (7), supporting previous empirical results (data not shown).

A search performed with the genome sequences obtained in this study in antibiotic resistance databases (8) did not detect putative genes involved in erythromycin and clindamycin resistance. Further studies must be conducted in order to know what genetic sequences encode the erythromycin/clindamycin resistance phenotype in *B. breve* CECT7263.

### Accession number(s).

The complete genome sequence has been deposited in GenBank under the accession number MWVR00000000 (BioProject no. PRJNA377846).
